# How people with knee pain understand why their pain changes or remains the same over time: A qualitative study

**DOI:** 10.1016/j.ocarto.2023.100345

**Published:** 2023-02-10

**Authors:** David A. Walsh, James Rathbone, Kehinde Akin-Akinyosoye, Gwen S. Fernandes, Ana M. Valdes, Daniel F. McWilliams, Weiya Zhang, Michael Doherty, Jennie E. Hancox, Kavita Vedhara, Roshan das Nair, Eamonn Ferguson

**Affiliations:** a Pain Centre Versus Arthritis, University of Nottingham, UK; b Academic Rheumatology, Division of Injury, Recovery and Inflammation Sciences, School of Medicine, University of Nottingham, UK; c NIHR Nottingham Biomedical Research Centre, Nottingham University Hospitals NHS Trust, UK; d Sherwood Forest Hospitals NHS Foundation Trust, Sutton in Ashfield, UK; e Division of Primary Care, School of Medicine, University of Nottingham, UK; f Mental Health & Clinical Neurosciences, School of Medicine, University of Nottingham, UK; g Department of Health Research, SINTEF, Trondheim, Norway; h School of Psychology, University of Nottingham, UK

**Keywords:** Pain-mechanisms, Pain-progression, Knee osteoarthritis, Anxiety, Beliefs

## Abstract

**Objectives:**

Guidelines recommend knee osteoarthritis pain management based on biopsychosocial mechanisms. Treatment adherence and effectiveness may be affected if there is a mismatch between patient perspectives and treatment focus. We therefore examined patient perspectives on mechanisms of their knee pain, why it persisted or changed over the past year, whether their understanding had changed, and whether their understanding aligned with that of others with whom they interact.

**Methods:**

Individuals with chronic knee pain (n ​= ​50) were purposively recruited from the Knee Pain and related health In the Community (KPIC) cohort to represent worsened, improved, or unchanged pain or anxiety between baseline and one year later. Framework analysis, a comparative form of thematic analysis, was used across transcripts of semi-structured telephone interviews.

**Results:**

Data were collapsed into themes of diagnosis, joint structure, ageing, physical activity, weight management, and treatment. Participants focused on biomechanical rather than psychological pain mechanisms. Some participants attributed pain improvement to increased and others to decreased physical activity. Participants reported no change in their understanding of their pain during the preceding year, but that their attitudes to pain, for example acceptance, had changed. Participants reported that they and others around them lacked understanding of their pain and why it did or did not change.

**Conclusion:**

People report a predominantly biomechanical understanding of why their knee pain remains constant or changes over time. Clinicians should support patients to develop a biopsychosocial understanding of knee pain aligned to treatment across the range of biological, psychological, and social modalities.

## 1 Introduction

One quarter of adults aged above 55 ​y experience significant knee pain, of whom half have radiographic osteoarthritis (OA) [1]. Heterogeneous mechanisms underlie knee pain's diverse qualities. OA pain is associated with pathology affecting articular cartilage, subchondral bone, synovium, and peri-articular tissues, and may be triggered or exacerbated by local biomechanical factors (peripheral mechanisms). Knee pain is also affected by central nervous system processing, associated with central sensitisation, cognition, anxiety and mood (central mechanisms) [2].

People with chronic pain often hold strong biomedical beliefs, attributing their pain to tissue damage [3,4]. Indeed, surgical replacement of a ‘damaged’ joint can be very successful in relieving pain. However, up to 20% continue to experience clinically important pain even after surgery. Chronic pain is both a sensory and emotional experience, and a biopsychosocial model has been widely adopted by research and healthcare communities [5]. This underpins currently recommended treatments for knee pain, including strengthening and mind-body exercise, peripheral and central-acting analgesics, surgery, and cognitive behavioural therapy [[6], [7], [8]]. Treatment uptake, efficacy and satisfaction require that beliefs are shared between patient and clinician [9].

Pain is a defining feature of OA for patients [10], but previous research has focused on beliefs about OA as a joint disease [4,11,12] Patients may attribute their OA to genes, being overweight, ageing, manual work, extensive kneeling or lifting, past sporting or occupational activities or trauma, or to unknown causes or chance [12]. Many people explain their OA as something caused by ‘wear and tear’ and loading of the joint, and believe that pain would be cured by replacing lost cartilage, or relieved by reducing joint loading [4,11,12]. Some believe that pain is caused by bone surfaces rubbing together, or that an increase in pain severity is due to structural progression to ‘bone on bone’ [4,11].

Scientific understanding of knee pain now incorporates many mechanisms beyond biomechanical factors in the joint. Increasing structural change might indeed be associated with increasing pain [13], although, for the individual, pain might improve irrespective of structural OA progression. Increases in pain may be associated also with anxiety or low mood, although, again, increasing distress is not inevitably accompanied by an increase in pain [14]. How people understand their knee pain might therefore often diverge from scientific evidence.

It is currently unknown how individuals make sense of their knee pain as it persists or changes over time, or whether their understanding might change as they experience changing pain. Inappropriate beliefs might contribute to chronic pain and be, of themselves, targets for interventions such as cognitive behavioural therapy [15]. Knowledge of how people understand their knee pain could identify information needs that, if addressed, might facilitate treatment and ultimately reduce the burden of chronic pain.

We conducted an interview-based study to investigate three topics from the perspective of individuals with knee pain: (1) their understanding of the mechanisms that drive their knee pain and whether their understanding converged with those of healthcare professionals and others with whom they interact; (2) their understanding of the reasons for persistent or changing pain over the past year; and (3) how their understanding had changed over the past year, regarding the mechanisms of their ongoing pain. We further sought to identify whether study findings were consistent across individuals with persistent or changing pain and anxiety.

## 2 Methods

### 2.1 Study design and procedures

This qualitative study used semi-structured interviews and a combined approach to framework analysis, a comparative form of thematic analysis, to develop themes both inductively from the views shared by the individual, and deductively from existing literature [16]. Ethical approval was from the University of Nottingham Faculty of Medicine and Health Sciences Research Ethics Committee (11–1704), the study was sponsored and received institutional approval from the University of Nottingham. All participants gave informed consent to the work. Interview guides were designed in partnership with individuals with knee pain from the Pain Centre Versus Arthritis Patient and Public Involvement and Engagement (PPIE) group. Concordance with the consolidated criteria for reporting qualitative research (COREQ) [17] is presented in Supplementary Table 1.

### 2.2 Participants

Participants were from the Knee Pain and Related Health in the Community Study (KPIC), a prospective cohort study designed to investigate mechanisms and associations of knee pain in community-dwelling adults [18]. KPIC is a sample of men and women aged 40 years and over, located on the UK General Practitioner (GP) register in the East Midlands, UK. KPIC recruited 9506 people at baseline irrespective of knee pain status, of whom 2512 reported pain in or around knee for most days in the past month. Of these, 1471 also completed questionnaires at the 1 year follow-up [18].

Eligibility for the current study required that KPIC participants had reported chronic knee pain at baseline by responding positively to: ‘Have you had knee pain on most days of this past month?’ and ‘Has your knee pain lasted more than 3 months?’ Eligibility also required completion of Numerical Rating Scores for pain and Hospital Anxiety and Depression Score (HADS) anxiety subscale [19] at baseline and 1-year follow up, and documented willingness to be contacted for research studies. Participants were excluded if they reported an inflammatory musculoskeletal condition (e.g., rheumatoid arthritis), reported at the 1-year KPIC follow-up acute knee pain (<3 months) attributed to trauma, or could not communicate in spoken English.

Invitations to participate and a participant information sheet were sent by post. We purposively sampled participants based on prospectively collected baseline and 1-year follow up KPIC questionnaire responses. To ensure that the study sample represented a range of individuals with persistent or changing knee pain and anxiety, interested participants were invited according to a 3 ​× ​3 ​cell recruitment matrix, representing all combinations of 3 levels of change (worsened, unchanged, or improved) for the 2 characteristics of pain or anxiety.

Participants were invited to interviews in waves, with progressive enrichment in successive waves of matrix groups that contained interviews from small numbers of participants from previous waves. Recruitment to interviews of KPIC participants in each study subgroup continued until a target number of interviews per group was reached (n ​= ​9), or further KPIC participants were not identified in that group. All interviews were conducted by one researcher (JR) and overall recruitment was discontinued when the researcher reported that no new information was emerging across study subgroups (data-saturation).

### 2.3 Data collection

Participant characteristics were determined using self-report questionnaire responses provided through KPIC [18]. At baseline, participants responded to questions ‘In the past 12 months, have you consulted a healthcare professional (e.g., your GP, a hospital specialist, a physiotherapist, etc.) about your knee pain?’ [Yes/No], and ‘Please list all your current medication including those prescribed by your doctor and those you bought yourself over the counter’ [free text]. The Intermittent and Constant OA Pain (ICOAP) questionnaire assessed the severity of intermittent and constant knee pain at baseline and at year 1 [20].

Pain change classification was determined at screening by response at year 1 to the question: ‘Since it has started, do you think the severity of your knee pain has overall … ‘; improved, remained the same, or worsened, AND a concordant change between baseline and year 1 follow up of at least 1 point on a numerical rating scale responding to the question ‘In the past month, how intense was your ‘worst knee pain’ rated on a 0–10 scale, where 0 is ‘no pain’ and 10 is ‘pain as bad as could be’?’ A 1-point change was selected to approximate to a minimal clinically important difference (MCID) [21]. Anxiety change classification was determined at screening according to a change in anxiety score between baseline and year 1 of at least 1 MCID on the HADS-Anxiety subscale [19]. Before each interview, participants were asked if they agreed with their classification by the researchers into ‘pain improved’, ‘stable’ or ‘worsened’, and reclassified where necessary according to their current perception of pain change over the past year.

Telephone interviews were conducted by one pain researcher (JR) who was also a clinical psychologist. Only the researcher and participant were present during the interview. Interview guides addressed the three research topics, customised to each pain change subgroup (Supplementary files 1–3). For each subgroup, questions 1 to 4 addressed Topic 1 (participant's understanding of the mechanisms of their current knee pain), question 5 Topic 2 (participant's understanding of the reasons behind their changing or persistent pain over the past year), and question 6 Topic 3 (change over the past year in the participant's understanding of their knee pain). Open-ended questions were used to elicit the participant's beliefs; the interviewer was free to ask relevant follow-up questions to seek more in-depth answers, and participants were free to introduce new topics. The interviews were audio-recorded, and the interviewer collected field notes to inform the analytic process.

### 2.4 Data analysis

Participant characteristics are presented as proportions, mean (standard deviation) or median (interquartile range). Interviews were transcribed verbatim by an independent transcriber, imported to NVivo 12 software [22], and analysed using a ‘Framework Analysis’ approach [16]. One researcher (JR) conducted an initial read through while listening to the recording to ensure concordance. After this, a framework of themes and subthemes was created using inductive and deductive approaches applied to each of the 3 topics addressed across each of the 50 interviews (Supplementary files 2–4). To ascertain reliability, all data were coded by two independent coders from different backgrounds (JR; male, Clinical Psychologist, KAA; female, non-clinical postdoctoral scientist) [23]. Where there was inconsistency, the relevant code was more carefully described and operationalised via a discussion within the research group. The codes were then reviewed, and categories created from the recurring data in the interviews. Data from the transcripts that supported each category were summarised and organised into broader themes across participants, and in each of the 9 subgroups. Primary themes were identified along with sub-themes, resulting in a hierarchy. Each transcript was classified as representing or not representing each theme.

## 3 Results

### 3.1 Participant selection and characteristics

One thousand four hundred and thirty-seven KPIC participants with knee pain submitted questionnaires at year 1 follow up. Fifty-four participants agreed to complete telephone interviews. Transcripts from the 9 pain/anxiety interview subgroups each contained between 2 (pain and anxiety improved) and 10 (pain increased, anxiety unchanged) participants (Table 1). Enrolled participants were similar in age and BMI to the eligible pool (n ​= ​1471), but were more likely to be women (p ​= ​0.048). Interviews lasted approximately 15 ​min each (range ​= ​9–26 ​min).

**Table 1:** Participant demographics compared across the eligible recruitment pool (n ​= ​1471) and the study population (n ​= ​50), according to purposive sampling matrix.

	PAIN DECREASED	PAIN UNCHANGED	PAIN INCREASED	TOTAL
**ANXIETY IMPROVED**				
***KPIC population***	**n ​= ​109**	**n ​= ​55**	**n ​= ​213**	**n ​= ​377**
Female (n (%))	58 (53%)	33 (60%)	154 (72%)	245 (65%)
Age (mean (range) y)	64 (41–80)	60 (41–80)	62 (42–80)	62 (40–82)
BMI (median (IQR) kg/m^2^)	29.3 (26.0–32.9)	28.2 (24.9–30.7)	28.1 (24.8–32.9)	28.2 (25.1–32.9)
***Recruited***	**n ​= ​2**	**n ​= ​4**	**n ​= ​7**	**n ​= ​13**
Female (n (%))	2 (100%)	4 (100%)	5 (71%)	11 (85%)
Age (mean (range) y)	59 (50–68)	64 (57–67)	68 (61–76)	65 (50–76)
BMI (median (IQR) kg/m^2^)	31.0 (26.9–35.1)	28.8 (26.9–30.3)	28.1 (24.5–31.5)	28.2 (25.6–31.2)

**ANXIETY UNCHANGED**				
***KPIC population***	**n ​= ​120**	**n ​= ​95**	**n ​= ​285**	**n ​= ​500**
Female (n (%))	60 (50%)	44 (46%)	171 (60%)	275 (55%)
Age (mean (range) y)	64 (43–80)	63 (56–70)	63 (41–81)	63 (40–82)
BMI (median (IQR) kg/m^2^)	27.7 (24.8–30.9)	27.8 (25.3–32.3)	27.8 (24.7–31.5)	27.8 (24.9–31.2)
***Recruited***	**n ​= ​6**	**n ​= ​8**	**n ​= ​10**	**n ​= ​24**
Female (n (%))	5 (83%)	5 (63%)	6 (60%)	16 (67%)
Age (mean (range) y)	61 (43–76)	65 (56–78)	63 (55–73)	63 (43–78)
BMI (median (IQR)) kg/m^2^)	27.3 (24.3–31.5)	29.9 (27.4–31.06)	28.2 (25.9–34.9)	28.8 (25.9–31.3)
**ANXIETY WORSENED**				
***KPIC population***	**n ​= ​47**	**n ​= ​60**	**n ​= ​166**	**n ​= ​273**
Female (n (%))	33 (70%)	34 (57%)	101 (61%)	168 (62%)
Age (mean (range) y)	62 (41–79)	62 (42–80)	60 (41–80)	61 (40–81)
BMI (median (IQR) kg/m^2^)	27.9 (25.3–33.4)	28.5 (25.8–32.1)	27.3 (24.7–31.7)	27.5 (25.0–32.2)
***Recruited***	**n ​= ​4**	**n ​= ​4**	**n ​= ​5**	**n ​= ​13**
Female (n (%))	3 (75%)	4 (100%)	3 (60%)	10 (77%)
Age (mean (range) y)	66 (61–73)	67 (53–79)	66 (58–75)	13 (53–79)
BMI (median (IQR) kg/m^2^)	29.3 (24.8–31.6)	26.2 (23.0–28.4)	26.9 (25.7–31.1)	27.6 (25.5–30.1)

**TOTAL**				
***KPIC population***	**n ​= ​276**	**n ​= ​210**	**n ​= ​664**	**n ​= ​1471**
Female (n (%))	151 (55%)	111 (53%)	426 (64%)	876 (60%)
Age (mean (range) y)	64 (40–82)	62 (41–80)	62 (40–82)	62 (40–85)
BMI (median (IQR) kg/m^2^)	28.4 (25.4–32.4)	28.1 (25.3–31.9)	27.7 (24.7–30.0)	27.7 (24.9–31.6)
***Recruited***	**n ​= ​12**	**n ​= ​19**	**n ​= ​19**	**n ​= ​50**
Female (n (%))	10 (83%)	13 (81%)	14 (64%)	37 (74%)
Age (mean (range) y)	62 (43–76)	64.9 (53–79)	22 (55–76)	65 (43–79)
BMI (median (IQR) kg/m^2^)	29.2 (24.7–33.1)	28.6 (26.2–30.8)	28.1 (25.5–31.4)	28.2 (25.5–31.2)

Heterogeneity in recruited subgroup sizes reflects heterogeneity in subgroup sizes in the eligible population and reclassification of 8 participants based on responses at interview to questions on whether their pain had changed over the past year.

At baseline, the mean (SD) age was 65 (8) years and 37 (74%) participants were women. The mean BMI was 28.5 (4.0) kg/m^2^ at baseline, and 29.0 (5.7) kg/m^2^ at 1 year follow up. Twenty-nine participants (58%) reported that they had received a diagnosis of knee osteoarthritis from a health professional. Participants reported a range of knee pain severities, anxiety, and depressive symptoms (median (IQR) baseline ICOAP-Intermittent; 9 (4–14), ICOAP-Constant; 7 (2–11)), HADS-Anxiety 10 (8–11), and HADS-Depression 7 (4–9)). At 1 year follow up, 46 (92%) participants reported having contact with a healthcare professional for their knee pain at least once during the preceding year. Ten (20%) participants reported use of paracetamol (acetaminophen), 13 (26%) non-steroidal anti-inflammatory drug (NSAID) use, and 12 (24%) opioid use.

### 3.2 Thematic findings

Results are provided under the topics addressed within the interview guide (Supplementary file 1–3). The provided quotes were selected to contextualise, illustrate or clarify the identified themes. Quotes are transliterations from the interview-recordings, presented without substantive editing, so that they retain their cultural meanings. Ten themes were developed across the 3 interview topics (Table 2). Some themes were shared between topics. For example, structural change within the joint, and ageing were developed in both topic 1 (mechanisms of current knee pain) and topic 2 (reasons behind changing or persistent pain). Pain and anxiety subgroup allocation of individuals providing quotes are given. Data from transcripts that supported each theme were observed from interviews across all subgroups, except where indicated within the text.

**Table 2:** Themes developed across interview topics.

Topic	Themes
Mechanisms of current knee pain	Understanding of their knee pain diagnosis
	Changes occurring within the joint
	Barriers to understanding and support
Reasons behind changing or persistent pain over the past year	Ageing
	Structural changes
	Change or stability in their level of physical activity
	Treatments and Aids
	Weight Management
Change over the past year in understanding of knee pain	No change in understanding
	Change in attitude

### 3.3 Topic 1: Participant's understanding of the mechanisms of their current knee pain

**Theme 1 ‘Understanding of their knee pain diagnosis’:** Participants indicated a range of how well they understood their knee pain diagnosis. Several, particularly those with unchanged pain over the year, demonstrated difficulty discussing their diagnosis and what it meant for them.

“Well it’s arthritis I’m told … I don’t know a great deal about it to be absolutely honest. I know it seems to be bones and they used to grind as I went upstairs …” - Participant 8249 (Same Pain, Improved anxiety)

**Theme 2 ‘Structural changes occurring within the joint**’: Some participants, whether or not they had been given a diagnosis, found it difficult to explain what might be happening with their knee, and why they were having pain. Some, particularly those whose pain improved or worsened over the year, attributed their current knee pain to ongoing processes within the affected knee. Explanations focused on structural change, biomechanical factors, and ageing.

“Yes, it’s due to arthritis, wear and tear over the years. And then it’s aggravated obviously if you knock it, which is due to arthritis … Yeah, I mean it’s deterioration isn’t it, of the cartilage and all around the knee … Well I suppose, because it disintegrates the cartilage and everything around the knee. The bones are probably – I don’t know – grating together? And also the way you walk, as well, aggravates it.” – Participant 2727 (Improved pain/Same anxiety)

**Theme 3** ‘**Barriers to understanding and support**’: Questions addressing understanding of knee pain and associated mechanisms also elicited discussion of the level of support that participants received in managing their knee pain, and barriers to seeking support when the participant perceived that others did not understand. Some participants felt they had experienced a lack of support and explanation from healthcare professionals or indicated that professionals would not be able to help them to resolve their pain. This left them unsure of how best to manage their knee pain.

“Well, the doctors just said it’s arthritis. I’m absolutely terrified of my knee going … it does really get to you. I daren’t do anything hardly anymore … I can’t. I daren’t go to a gym myself, because they don’t know what’s wrong with me. I can say what’s wrong with me, but I don’t know if they would be able to help me.” Participant 4782 (Worse pain/Improved anxiety)

Participants discussed seeking support from friends and family, and some found this helpful. However, some participants explained that a lot of the people around them did not understand their pain or would offer unhelpful advice. Other participants felt they could not talk to others about their pain because they thought that if they did so they would be a burden, or they expressed beliefs that other people's problems were greater than their own.

“Oh, their view is it’s just my own silly fault for working too hard. Yeah. I wouldn’t score them highly on sympathy. Well, they are brief conversations and they always say ‘well, don’t do so much’. Well, that’s not an answer that I’m very happy with. We don’t talk about it now.” – Participant 786 (Same pain/Same anxiety)

### 3.4 Topic 2: Participant's understanding of the reasons behind their changing or persistent pain over the past year

Some participants, during the early part of their interview, expressed uncertainty about why their knee pain had or had not changed in the past year.

“I just don’t know. I’m at a loss about it now really.” – Participant 1418 (Same Pain/Worse anxiety)

Despite further probing, some with increasing or decreasing pain could not provide any reasons for why their knee pain had changed over the past year.

**Theme 4 ‘Age’:** Some participants attributed their changing pain to age. None within any of the improved pain subgroups discussed that age contributed to their changing pain.

“No I just thought that it comes with age.” – Participant 1177 (Worse Pain/Improved anxiety)

**Theme 5 ‘Structural changes within the affected joint**’: Some participants across all 9 subgroups attributed their changing pain to ongoing structural changes within the affected joint.

“I do still think it’s soft tissue and I think there might be some kind of early arthritis but it’s so early that there wouldn’t be any bone changes it would just be like soft tissue” – Participant 3280 (Worse pain/Worse anxiety)

**Theme 6 ‘Change in physical activity’:** Some participants across all 9 subgroups explained that their change or no change in knee pain during the past year was due to a change or lack of change in their level of physical activity. Different participants displayed different perspectives on the positive or negative impact of physical activity on changing pain over the past year. Some individuals discussed that increased physical activity and exercise led to pain improvements, whereas others indicated that their pain was reduced due to reduced physical activity or due to resting.

“I think because I took exercise, mainly on things like the treadmill – but not like crazy stuff, but just pacing myself and kept it [knee] strong, then I think I kept it [knee pain] at bay.” – Participant 5780 (Same pain/Same anxiety)

**Theme 7 ‘Treatments and Aids’**: Some participants attributed changes in pain over the past year to treatments (e.g., analgesic medications, knee replacement surgery), or aids (e.g., walking sticks). However, no participant who showed improvements in pain or anxiety over the past year attributed their changing pain levels to analgesic use.

“I think it’s because … I take my medication [anti-inflammatory]. Even if I’m not in pain, I know I’ve got to take that medication. You know, I know that I need it. I know when I haven’t taken it, when I’ve forgotten to take it, I do know.” - Participant 5165 (Same pain/Worse anxiety).

**Theme 8 ‘Weight management’**: Weight management was discussed by individuals who indicated a belief that keeping weight down was beneficial for pain relief. None of the participants who had worsened pain or anxiety over the past year contributed to the theme of weight management.

“I physically feel better for having lost weight which I think will help my knees, it will also help my hip.” – Participant 8765 (Improved pain/Same anxiety)

### 3.5 Topic 3: change over the past year in the participant's understanding of their knee pain

**Theme 9 ‘No change in understanding’**: Participants did not describe a change in understanding of their pain regardless of whether it had improved, remained the same or worsened. Some participants explained that their understanding had not changed because they had not engaged with further care for their knee pain. Whilst some had received a diagnosis of arthritis in the year between assessment points, it was felt this only confirmed what they already believed to be true.

“I think my understanding has stayed the same, mainly because I haven’t looked into it any further, and because I haven’t had any major flare-ups. So I haven’t needed to change my understanding.“- Participant 5780 (Same pain/Same anxiety)

**Theme 10 ‘Change in attitude’**: Some explained that they had changed their attitude, rather than their understanding of knee pain.

“I think that I’ve just accepted what the Consultant said and I’ve got to put up with it. It’s one of those things. There’s nothing, there’s no magic wand to cure it … but with me like I can’t have it [Knee Replacement Surgery] so that I’ve got to accept it you know.” – Participant 5341 (Worse pain, Worse anxiety)

## 4 Discussion

We found that participants in a community cohort reported that they lacked understanding of their knee pain and why it does or does not change over time. People explained their knee pain predominantly according to biomechanical mechanisms linked to diagnosis, joint structure, ageing, physical activity, weight management, and treatment. Interpretations varied little across pain and anxiety subgroups, and interpretations could be contradictory between individuals. For example, some participants attributed pain improvement to increased activity, whereas others in the same subgroup attributed their pain improvement to decreased physical activity. Participants reported changes in their attitudes which manifested as a general acceptance of their condition, but not changes in understanding of their pain during the preceding year. Our findings suggest that a biopsychosocial understanding of knee pain might not be shared by people in the community who experience knee pain. Participants in our study displayed insight into the incompleteness of their understanding of knee pain mechanisms, suggesting an openness to interventions that could help develop concordant understanding across society.

Previous research has considered how people perceive OA as an illness, recognising pain as a key symptom attributable to OA [12,24]. However, how people make sense of mechanisms by which OA causes pain is less completely understood. Our participants described pain mechanisms closely aligned to reported understanding of joint pathology; being overweight, ageing, manual work, extensive kneeling or lifting, past sporting activities trauma, structural deterioration worn away by movement or to unknown causes [12]. We used stratified recruitment based both on changing pain and changing anxiety, considering chronic pain as both a sensory and emotional experience [20]. However, we found that participants’ understanding of their pain attributed little to psychological mechanisms, and interpretations varied little across subgroups with reducing, stable, or increasing HADS-anxiety scores. A biomechanical understanding of OA as a disease seems to translate into a biomechanical understanding of OA pain. Reciprocally, people may interpret a change in pain as indicating structural change [11].

Poor patient understanding of OA might be a key treatment barrier [25]. A biomechanical understanding supports some evidence-based interventions to relieve knee pain such as strengthening exercises, orthotics, and arthroplasty. However, people who believe OA to represent ‘bone on bone’ or a ‘worn out’ joint might be more likely to seek surgical joint replacement, rather than non-surgical interventions such as exercise [11]. A predominantly biomechanical understanding might associate worsening knee pain, for example during exercise, with ongoing joint damage, encouraging catastrophic beliefs which in turn exacerbate pain and pose barriers to effective treatment [26,27]. Our participants scarcely discussed psychological pain mechanisms, central sensitisation or descending inhibition, despite scientific evidence that these might be effective analgesic targets. Changing patient perceptions and understanding could facilitate uptake of non-pharmacological interventions among individuals with OA [28].

Participants in our study indicated that their biomechanical beliefs about knee pain were based on previous engagement with healthcare professionals, and that limited information from their healthcare team served as a barrier to understanding their diagnosis. Perceived deficiencies in understanding might predict future increases in disability, and worsen over time in those with increasing disability [24]. Clinical guidelines recommend that clinicians ‘offer accurate verbal and written information to all people … to enhance understanding of the condition and its management, and to counter misconceptions’ [8]. Healthcare teams might unlock an unrealised potential for individualised information and shared decision-making with patients to promote understanding, and ultimately pain relief [29].

Our study had some limitations. Interviews were conducted at a single time point, limiting interpretation of participants’ perception of their changing understanding of pain. Our study might have been subject to misclassification bias due to the variable nature of knee pain. Whereas our study had an appropriate overall sample size, some subgroups were small, reflecting their relative rarity even within the large KPIC population. Larger studies might identify more diverse mechanistic beliefs amongst people whose pain and anxiety are both improved. Changes in understanding of OA pain mechanisms might occur over a longer time frame than explored in the current study. In one study, people with greater progression of disability over 6 years reported a decrease in understanding about their OA, whereas others reported improved understanding [24]. Themes identified in this study depend in part on the perspective of the researchers, and different researchers might identify alternative or additional themes.

In conclusion, individuals with knee pain in the community report a predominantly biomechanical understanding of why their knee pain remains constant or changes over time. In contrast to biopsychosocial models of chronic pain adopted by researchers and healthcare professionals, psychological factors might contribute little to the sense that people make of their pain. Healthcare practitioners might be in an ideal position to promote a holistic and multifaceted model of pain and influence patients’ understanding of pain mechanisms. A greater understanding of pain mechanisms, and how current evidence-based treatments might work to improve knee pain outcomes, could facilitate engagement and convergence both with biomechanical and with psychological interventions.

## Author contributions

Conception of study DAW, JR, KAA, KV, RdN. Study design, acquisition of data or analysis and interpretation of data: DAW, JR, KAA, GSF, AMV, DFM, WZ, MD, JEH, KV, RdN, EF. Drafting the article, revising for critically important information: DAW, KAA, GSF, AMV, DFM, WZ, MD, JEH, KV, RdN, EF. Final approval of version submitted: DAW, JR, KAA, GSF, AMV, DFM, WZ, MD, JEH, KV, RdN, EF.

## Role of the funding source

This work was supported by 10.13039/501100000837University of Nottingham as Sponsor and host institution and by Sherwood Forest Hospitals NHS Foundation Trust as principal research site. This research was funded by 10.13039/501100012041Versus Arthritis (Centre initiative grant number 20777) and by the 10.13039/501100000272National Institute for Health Research through the 10.13039/501100000272NIHR Nottingham Biomedical Research Centre. The views expressed are those of the author(s) and not necessarily those of the NHS, Versus Arthritis, NIHR or the Department of Health and Social Care. Funders and study sponsor had no role in the study design, collection, analysis and interpretation of data; in the writing of the manuscript; and in the decision to submit the manuscript for publication.

## Declaration of competing interest

DAW declares a personal financial interest in his employment by the 10.13039/501100000837University of Nottingham who receive funding for his salary from the 10.13039/100013986UK Government, Sherwood Forest Hospitals NHS Foundation Trust and 10.13039/100014013UKRI. DAW declares non-personal financial interests in consultancy through his employment with the 10.13039/501100000837University of Nottingham to Pfizer Ltd, Eli Lilly, AbbVie Ltd, Galapagos and Reckitt Benckiser Health Ltd, AKL Research and Development Ltd, and 10.13039/100004330GlaxoSmithKline Plc, and responsibilities for investigator-led grants outside this work by the 10.13039/501100000837University of Nottingham from Pfizer, Eli Lilly and UCB Biopharma UK. RdN has received Speakers' Bureau payments from Biogen, Novartis, and Merck. DMcW declares a personal financial interest in his employment by the 10.13039/501100000837University of Nottingham, who receive funding for his salary from 10.13039/100011330Nottingham University Hospitals NHS Trust and 10.13039/501100000272National Institute for Health and Care Research (NIHR), and non-personal financial interests in investigator-led grants outside this work held by the 10.13039/501100000837University of Nottingham from Pfizer, Eli Lilly and UCB Biopharma UK. WZ declares personal financial interests outside this work in Advisory Board memberships for Eli Lilly and Regeneron, and Speaker Bureaus for Harbin Rheumatology Society and 10.13039/501100004791Shenzhen Rheumatology and Inflammation Forum, and non-personal financial interests in investigator-led grants outside this work held by the 10.13039/501100000837University of Nottingham from Foundation for Research in Rheumatology (FOREUM), Football Association (FA) and the Professional Footballers’ Association (PFA). EF declares personal financial interests outside this work in royalties or licenses from GL Assessment for the Paediatric Index of Emotional Distress (PI-ED), and consultancy for 10.13039/501100000780European Union, and non-personal financial interests in investigator-led grants outside this work held by the 10.13039/501100000837University of Nottingham from 10.13039/501100012041Versus Arthritis, 10.13039/100014013UKRI, 10.13039/501100013589Research England, 10.13039/501100000272NIHR, 10.13039/501100000286British Academy, 10.13039/100009033NHSBT Trust Fund, Pfizer, 10.13039/501100009187Medical Research Foundation, FA and PFA.
