# Supplementary material for: How people with knee pain understand why their pain changes or remains the same over time: A qualitative study
Source: Osteoarthr Cartil Open. 2023 Feb 10;5(2):100345. doi: 10.1016/j.ocarto.2023.100345 (PMC9958477; doi:10.1016/j.ocarto.2023.100345)
Supplement: Multimedia component 1 [file mmc1.docx]

# Supplementary Tables

## Supplementary Table 1: COREQ (COnsolidated criteria for REporting Qualitative research) Checklist

| **Topic** | **Item No.** | **Guide Questions/Description** | **Reported on Page No.** |
| --- | --- | --- | --- |
| **Domain 1: Research team**  **and reflexivity** | | | |
| *Personal characteristics* | | | |
| Interviewer/facilitator | 1 | Which author/s conducted the interview or focus group? | Dr James Rathbone conducted the interviews (Page 6) |
| Credentials | 2 | What were the researcher’s credentials? E.g. PhD, MD | DClinPsych (Page 6) |
| Occupation | 3 | What was their occupation at the time of the study? | Pain researcher and Clinical Psychologist (Page 6) |
| Gender | 4 | Was the researcher male or female? | Male (Page 6) |
| Experience and training | 5 | What experience or training did the researcher have? | Doctorate training and years of research experience (Page 6) |
| *Relationship with*  *participants* | | | |
| Relationship established | 6 | Was a relationship established prior to study commencement? | Participants were initially contacted by post from a research database linked to a previous study (KPIC) and were not known to the researcher prior to study commencement (page 5). |
| Participant knowledge of the interviewer | 7 | What did the participants know about the researcher? e.g. personal goals, reasons for doing the research | Participants only knew of the study aims and were not informed about any of the researcher’s personal goals (Supplementary files 1 - 3) |
| Interviewer characteristics | 8 | What characteristics were reported about the interviewer/facilitator? e.g. Bias, assumptions, reasons and interests in the research topic | Concepts that drove the interviewer’s assumptions for the study are described (page 5). |
| **Domain 2: Study design** | | | |
| *Theoretical framework* | | | |
| Methodological orientation and Theory | 9 | What methodological orientation was stated to underpin the study? e.g. grounded theory, discourse analysis, ethnography, phenomenology, content analysis | Thematic framework analysis (Page 5 and 7) |
| *Participant selection* | | | |
| Sampling | 10 | How were participants selected? e.g. purposive, convenience, consecutive, snowball | Purposive (page 5) |
| Method of approach | 11 | How were participants approached? e.g. face-to-face, telephone, mail, email | Participants were approached by post (page 5) |
| Sample size | 12 | How many participants were in the study? | 50 participants (page 7) |
| Non-participation | 13 | How many people refused to participate or dropped out? Reasons? | Transcripts from 4 participants were excluded from analysis due to; incomplete baseline or year 1 data (n=2), unclear audio-recording (n=1), or self-report of inflammatory arthritis during the interview (n=1). (Page 7) |
| *Setting* | | | |
| Setting of data collection | 14 | Where was the data collected? e.g. home, clinic, workplace | Participants were interviewed over the phone while at home (page 6) |
| Presence of non  participants | 15 | Was anyone else present besides the participants and researchers? | None known or heard to contribute during interviews (page 6) |
| Description of sample | 16 | What are the important characteristics of the sample? e.g. demographic data, date | Page 7 |
| *Data collection* | | | |
| Interview guide | 17 | Were questions, prompts, guides provided by the authors? Was it pilot tested? | Questions, prompts and guides employed within the current study are provided in Supplementary files 1-3. Interview guides were pilot tested by individuals with knee pain (page 5). |
| Repeat interviews | 18 | Were repeat interviews carried out? If yes, how many? | No repeat interviews were carried out |
| Audio/visual recording | 19 | Did the research use audio or visual recording to collect the data? | Audio recordings of the interviews were made (page 6) |
| Field notes | 20 | Were field notes made during and/or after the interview or focus group? | Yes, field notes were recorded at the time of the interview (page 7) |
| Duration | 21 | What was the duration of the interviews or focus group? | Interviews lasted approximately 15 minutes each (range = 9 to 26 minutes). (page 7) |
| Data saturation | 22 | Was data saturation discussed? | Yes. Data saturation was discussed and decided on by the research team (page 7) |
| Transcripts returned | 23 | Were transcripts returned to participants for comment and/or correction? | No. Transcripts were not shared with study participants |

| **Domain 3: analysis and**  **Findings** | | | |
| --- | --- | --- | --- |
| *Data analysis* | | | |
| Number of data coders | 24 | How many data coders coded the data? | 2 researchers (JR and KAA) coded the data independently (page 7) |
| Description of the coding tree | 25 | Did authors provide a description of the coding tree? | A description of the coding tree, according to the topic investigated in the study, is described in page 7. |
| Derivation of themes | 26 | Were themes identified in advance or derived from the data? | A hybrid inductive and deductive approach was employed during data analysis (page 5) |
| Software | 27 | What software, if applicable, was used to manage the data? | N-Vivo was used to manage and analyse the study data (page 7) |
| Participant checking | 28 | Did participants provide feedback on the findings? | No participant feedback on the findings were obtained or reported in this study. |
| *Reporting* | | | |
| Quotations presented | 29 | Were participant quotations presented to illustrate the themes/findings? Was each quotation identified? e.g. participant number | Participant quotations were illustrative of each identified theme and subtheme (pages 8 - 11).  Each quotation was identified by the encrypted participant number. |
| Data and findings consistent | 30 | Was there consistency between the data presented and the findings? | Yes, the data presented and the findings were consistent (page 8 - 11) |
| Clarity of major themes | 31 | Were major themes clearly presented in the findings? | Each theme and subtheme is presented clearly within their respective sections (pages 8 - 11). |
| Clarity of minor themes | 32 | Is there a description of diverse cases or discussion of minor themes? | Yes. Where minor trends were observed, these were reported in the results section (page 8 - 11). |

Developed from: Tong A, Sainsbury P, Craig J. Consolidated criteria for reporting qualitative research (COREQ): a 32-item checklist for interviews and focus groups. *International Journal for Quality in Health Care*. 2007. Volume 19, Number 6: pp. 349 – 357

# Supplementary file 1: Interview guide for worsened pain groups


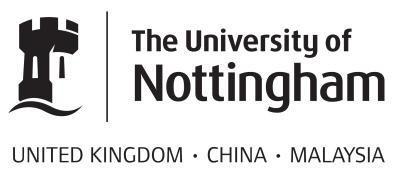


**Interview schedule**

START CALL

**Introduction**

Hello, my name is James Rathbone and I’m a researcher with Arthritis Research UK at the University of Nottingham. I’m calling because you have sent back a consent form saying you are willing to take part in some research looking at understanding of knee pain over time.

*Were you expecting this call today?*

I would like to know a little more about your knee pain. When we looked at your responses on the Knee Pain in the Community questionnaire it highlighted that your knee pain had got worse over the time from when you first filled it out to one year later. I’m going to ask you some questions about your understanding of your pain and how it has changed.

The telephone call will only take 15-20 minutes depending on how much you would like to say in answer to each question. There are 6 questions in total although some may have a follow-up question as well.

Before we start I just have a few things to tell you;

As you will be aware from the information you received about the study I will be recording this telephone call. We are audio recording all of our interviews to help with data analysis once all the interviews have all taken place.

*The recording equipment is running and I will let you know when it has been switched off. Are you happy with this?*

Secondly, there are no right or wrong answers to the questions I will ask you. I would like you to answer them based on your real experiences of knee pain. If any of your answers result in me becoming concerned about your safety or the safety of others then I may need to share this information with other authorities. For example, this could be your GP. However, I will talk with you some more about this should the need arise.

Finally, if you would like to withdraw from the study and stop the interview at any time then you can do so without giving a reason.

*Do you have any questions for me?*

*Are you happy to go ahead with the interview today?*

Okay, let’s begin with the first question;

**Questions**

1. Can you tell me what you understand about the cause of your current knee pain?

*(Prompt; why do you think your knee is painful? What do you think is causing your knee pain? Why do you think you still have knee pain now?)*

1. Are there any other things that you can think of that play an important role in the amount of knee pain you experience?

*(Prompt; what makes your knee pain worse or better?)*

1. Do you think that the health professionals that have been involved in your care; for example, your GP, physiotherapist or hospital doctors, share your view on the cause of your pain?

*(Prompt; do health professionals think there is a different reason for your knee pain to you?)*

1. What about other people; for example your friends, family, or employer?

*(Prompt; do you friends, family, or employer think there is a different reason for your knee pain to you?)*

1. Why do you think your knee pain has got worse this past one year period?

*(Prompt; are there any other reasons why your knee pain may have got worse over the past one year period?)*

1. Has your understanding of your pain changed over this period?
2. If so, what has changed?
3. What caused this change in understanding?

Thank you! That was my final question. I will now switch off the audio recording equipment.

Okay, it’s now switched off and the call is no longer being recorded.

Thank you for participating in this study.

Do you have any questions for me about the study or the interview?

END CALL

# **Supplementary file 2: Interview guide for improved pain groups**


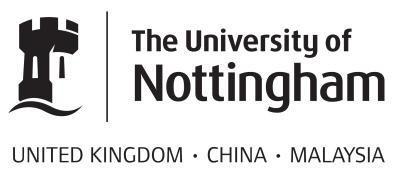


**Interview schedule**

START CALL

**Introduction**

Hello, my name is James Rathbone and I’m a researcher with Arthritis Research UK at the University of Nottingham. I’m calling because you have sent back a consent form saying you are willing to take part in some research looking at understanding of knee pain over time.

*Were you expecting this call today?*

I would like to know a little more about your knee pain. When we looked at your responses on the Knee Pain in the Community questionnaire it highlighted that your knee pain had improved over the time from when you first filled it out to one year later. I’m going to ask you some questions about your understanding of your pain and how it has changed.

The telephone call will only take 15-20 minutes depending on how much you would like to say in answer to each question. There are 6 questions in total although some may have a follow-up question as well.

Before we start I just have a few things to tell you;

As you will be aware from the information you received about the study I will be recording this telephone call. We are audio recording all of our interviews to help with data analysis once all the interviews have all taken place.

*The recording equipment is running and I will let you know when it has been switched off. Are you happy with this?*

Secondly, there are no right or wrong answers to the questions I will ask you. I would like you to answer them based on your real experiences of knee pain. If any of your answers result in me becoming concerned about your safety or the safety of others then I may need to share this information with other authorities. For example, this could be your GP. However, I will talk with you some more about this should the need arise.

Finally, if you would like to withdraw from the study and stop the interview at any time then you can do so without giving a reason.

*Do you have any questions for me?*

*Are you happy to go ahead with the interview today?*

Okay, let’s begin with the first question;

**Questions**

1. Can you tell me what you understand about the cause of your current knee pain?

*(Prompt; why do you think your knee is painful? What do you think is causing your knee pain? Why do you think you still have knee pain now?)*

1. Are there any other things that you can think of that play an important role in the amount of knee pain you experience?

*(Prompt; what makes your knee pain worse or better?)*

1. Do you think that the health professionals that have been involved in your care; for example, your GP, physiotherapist or hospital doctors, share your view on the cause of your pain?

*(Prompt; do health professionals think there is a different reason for your knee pain to you?)*

1. What about other people; for example your friends, family, or employer?

*(Prompt; do you friends, family, or employer think there is a different reason for your knee pain to you?)*

1. Why do you think your knee pain has improved this past one year period?

*(Prompt; are there any other reasons why your knee pain may have improved over the past one year period?)*

1. Has your understanding of your pain changed over this period?
2. If so, what has changed?
3. What caused this change in understanding?

Thank you! That was my final question. I will now switch off the audio recording equipment.

Okay, it’s now switched off and the call is no longer being recorded.

Thank you for participating in this study.

Do you have any questions for me about the study or the interview?

END CALL

# **Supplementary file 3: Interview guide for no change in pain groups**



**Interview schedule**

START CALL

**Introduction**

Hello, my name is James Rathbone and I’m a researcher with Arthritis Research UK at the University of Nottingham. I’m calling because you have sent back a consent form saying you are willing to take part in some research looking at understanding of knee pain over time.

*Were you expecting this call today?*

I would like to know a little more about your knee pain. When we looked at your responses on the Knee Pain in the Community questionnaire it highlighted that your knee pain had stayed the same over the time from when you first filled it out to one year later. I’m going to ask you some questions about your understanding of your pain.

The telephone call will only take 15-20 minutes depending on how much you would like to say in answer to each question. There are 6 questions in total although some may have a follow-up question as well.

Before we start I just have a few things to tell you;

As you will be aware from the information you received about the study I will be recording this telephone call. We are audio recording all of our interviews to help with data analysis once all the interviews have all taken place.

*The recording equipment is running and I will let you know when it has been switched off. Are you happy with this?*

Secondly, there are no right or wrong answers to the questions I will ask you. I would like you to answer them based on your real experiences of knee pain. If any of your answers result in me becoming concerned about your safety or the safety of others then I may need to share this information with other authorities. For example, this could be your GP. However, I will talk with you some more about this should the need arise.

Finally, if you would like to withdraw from the study and stop the interview at any time then you can do so without giving a reason.

*Do you have any questions for me?*

*Are you happy to go ahead with the interview today?*

Okay, let’s begin with the first question;

**Questions**

1. Can you tell me what you understand about the cause of your current knee pain?

*(Prompt; why do you think your knee is painful? What do you think is causing your knee pain? Why do you think you still have knee pain now?)*

1. Are there any other things that you can think of that play an important role in the amount of knee pain you experience?

*(Prompt; what makes your knee pain worse or better?)*

1. Do you think that the health professionals that have been involved in your care; for example, your GP, physiotherapist or hospital doctors, share your view on the cause of your pain?

*(Prompt; do health professionals think there is a different reason for your knee pain to you?)*

1. What about other people; for example your friends, family, or employer?

*(Prompt; do you friends, family, or employer think there is a different reason for your knee pain to you?)*

1. Why do you think your knee pain has stayed the same over this past one year period?

*(Prompt; are there any other reasons why your knee pain may have stayed the same over the past one year period?)*

1. Has your understanding of your pain changed over this period?
2. If so, what has changed?
3. What caused this change in understanding?

Thank you! That was my final question. I will now switch off the audio recording equipment.

Okay, it’s now switched off and the call is no longer being recorded.

Thank you for participating in this study.

Do you have any questions for me about the study or the interview?

END CALL
